# Computed tomography imaging of subpleural lipoma in two men: two case reports

**DOI:** 10.1186/1752-1947-4-380

**Published:** 2010-11-25

**Authors:** Christoph A Karlo, Paul Stolzmann, Thomas Frauenfelder, Olivio F Donati, Sebastian Leschka

**Affiliations:** 1 Institute for Diagnostic Radiology, University Hospital Zurich, Zurich, Switzerland

## Abstract

**Introduction:**

Subpleural lipomas are very rare pleural lesions and are often discovered incidentally in asymptomatic patients on conventional radiographs.

**Case presentations:**

We report two cases of subpleural lipomas and describe their imaging characteristics on chest radiographs and computed tomography. We describe the cases of two Caucasian men, aged 77 and 62 years old.

**Conclusions:**

For non-invasive diagnostic investigation, computed tomography enables the identification and quantification of these tumors due to their characteristic fat attenuation.

## Introduction

Lipomas in general are encapsulated, homogeneous, mesenchymal, slow growing tumors that arise in adipose tissue. Although they account for the most common benign soft tissue tumors in humans, intra-thoracic lipomas are considered rare. Subpleural lipomas are parenchymal, fat-containing lesions of the chest, where they arise from the submesothelial layers of the pleura parietalis and may extend into the subpleural, pleural or extrapleural space [1]. Most patients remain asymptomatic and the lipoma is found incidentally on a chest radiograph or a computed tomography (CT) examination. Since only a few cases have been published in the recent literature, we wanted to report two cases of subpleural lipomas with imaging findings from chest radiographs and CT examinations.

## Case presentations

### Case 1

**Figure 1:**
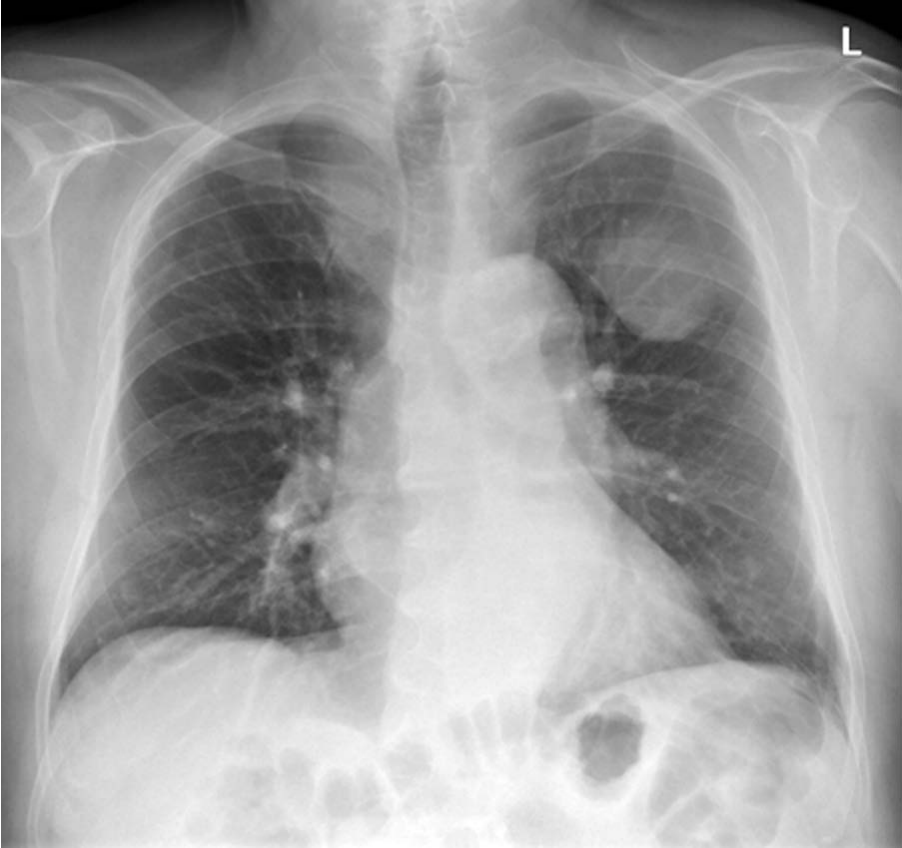
**Posterior-anterior chest radiograph demonstrating a well delineated, solid mass in projection to the left upper lobe in our 77-year-old patient**. Note the angle between the lesion's border and the pleura, which suggests the lesion to be of pleural origin.

**Figure 2:**
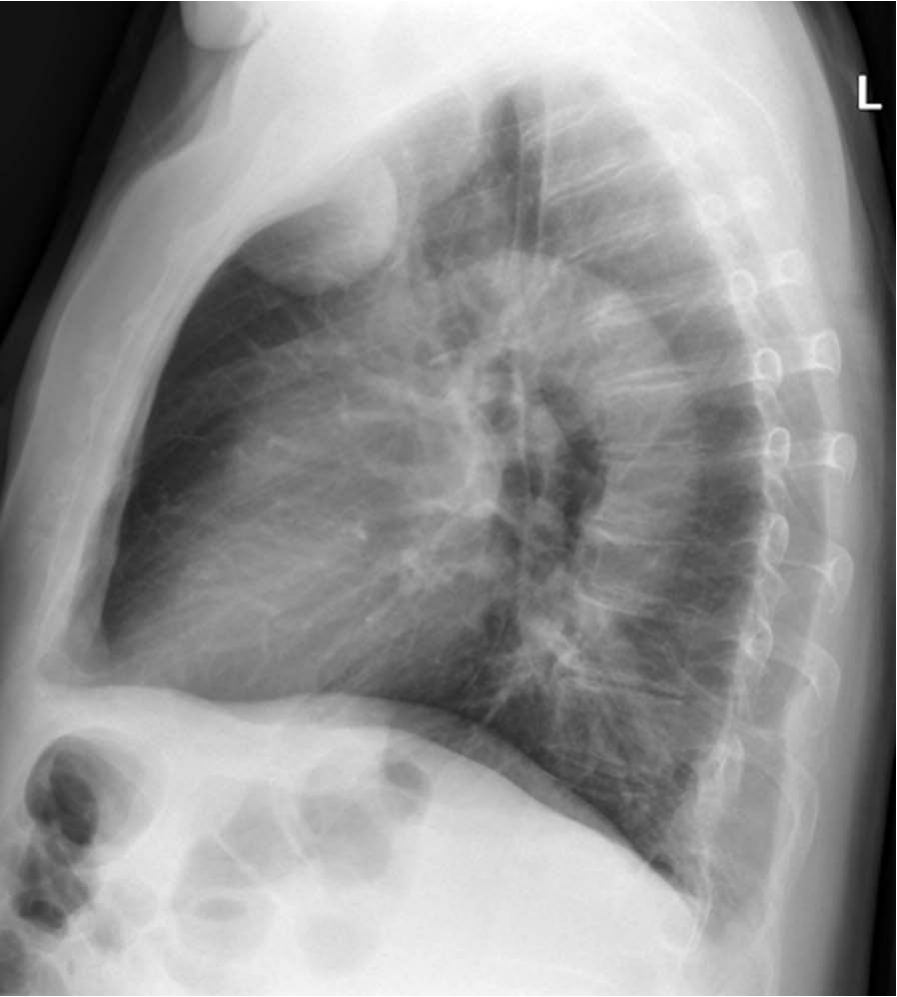
**Lateral view chest radiograph demonstrating the well delineated, solid mass in projection to the left upper lobe adjacent to the ventral chest wall in our 77-year-old patient**.

**Figure 3:**
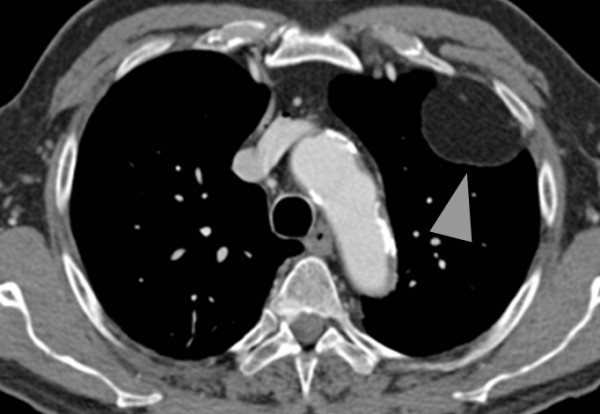
**Axial computed tomography (CT) image showing the characteristic hypodense condition of the lesion compatible with a fat-containing tumor, in this case a subpleural lipoma**.

**Figure 4:**
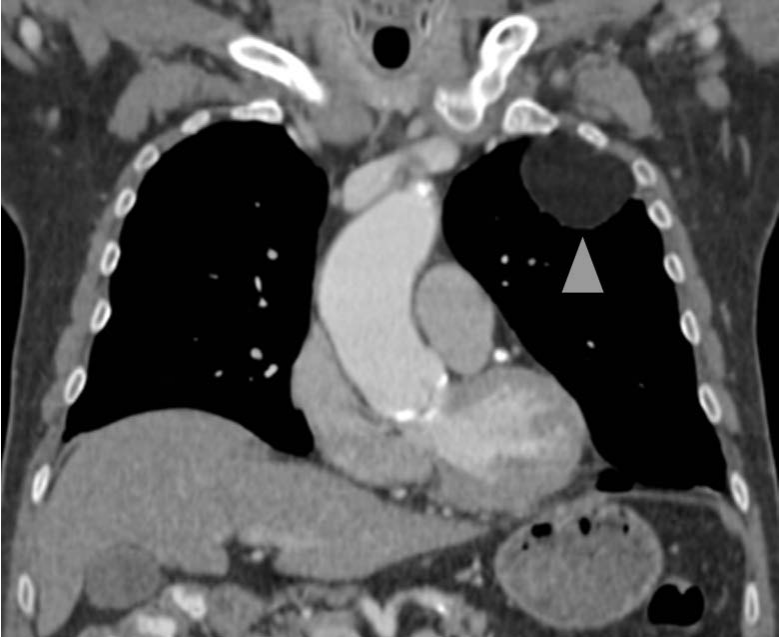
**Coronal reformatted computed tomography (CT) image showing the subpleural lipoma (arrowhead)**.

A 77-year-old Caucasian man was referred to our hospital with a three-month history of recurring chest pain, increasing in intensity over the last few days. He was a smoker and suffered from high blood pressure, however he has refused antihypertensive medication since diagnosis. In 2006, he underwent percutaneous, transluminal angioplasty of his left leg for a high-grade stenosis of the left femoral artery due to peripheral artery occlusive disease. No other significant events were found in his medical history. At a multidisciplinary team meeting, the decision to refer our patient for coronary catheter angiography was made because of his clinical findings and high pre-test probability for coronary artery disease. Before our patient underwent catheterization, upright posterior-anterior and lateral view chest radiographs were taken (Figure 1 and 2) and a solid, well delineated lesion in the left upper lobe was found. For further evaluation of this lesion, our patient was referred for CT, where a subpleural lipoma was diagnosed (Figure 3 and 4). Since this tumor did not show any signs of malignancy such as infiltration of the adjacent lung or chest wall structures or contrast enhancement, our patient went on to undergo catheter angiography and eventually underwent cardiac surgery for two aortocoronary bypass grafts.

### Case 2

**Figure 5:**
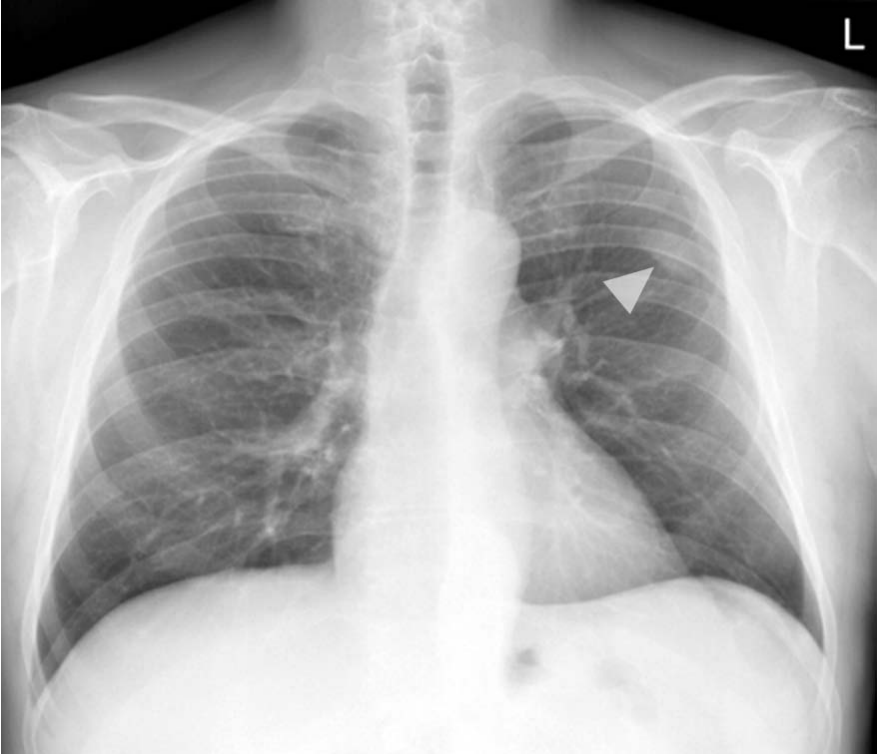
**Posterior-anterior view chest radiograph demonstrating a small, well delineated, solid lesion (arrowhead) in projection to the upper lobe on the left side in our 62-year-old patient**.

**Figure 6:**
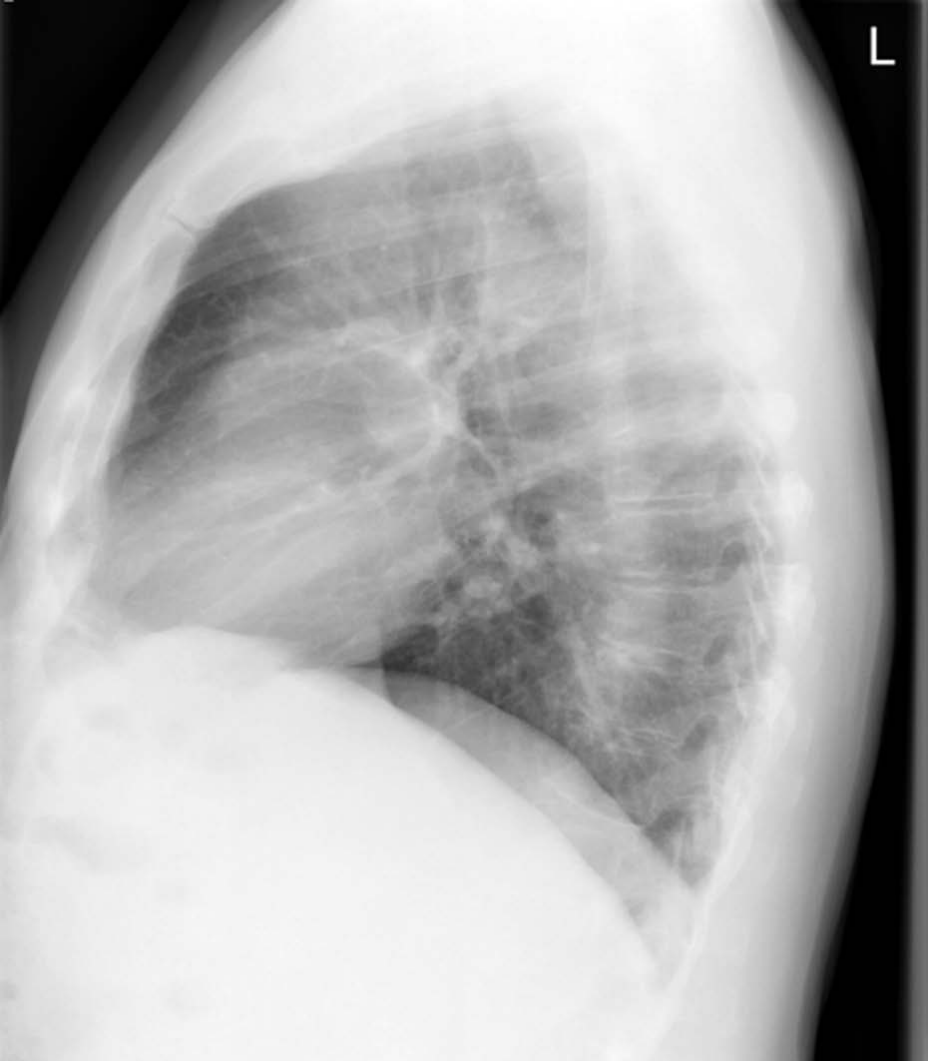
**The small, well delineated, solid lesion shown on Figure 5 was not detectable on the lateral view chest radiograph**.

**Figure 7:**
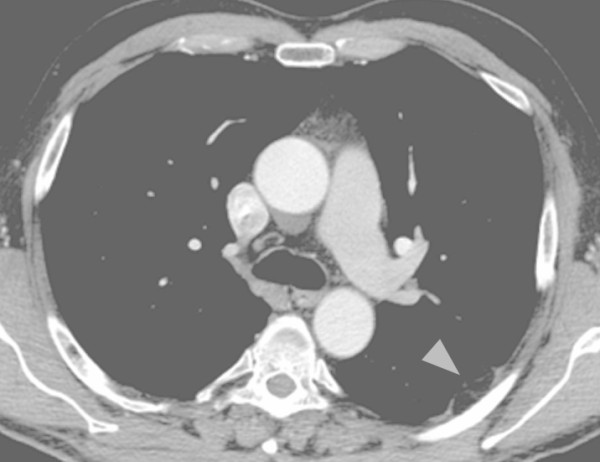
**Axial computed tomography (CT) image in mediastinal soft tissue window-level setting shows the characteristic appearance of a fat-containing tumor with density values of approximately -50 to -100HU (Hounsfield units), in this case a subpleural lipoma**.

**Figure 8:**
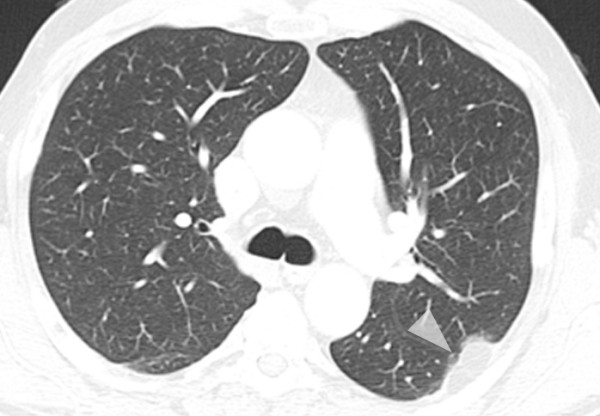
**Axial computed tomography (CT) image in lung window-level setting depicting the subpleural lipoma**.

A 62-year-old Caucasian man was referred to our hospital by his general practitioner for a routine, annual follow-up examination after he had undergone surgery for two plasmacytoma lesions in the epipharynx nine years ago. He had been in complete remission since then, however his general practitioner requested a chest radiograph because of a three-month history of intermittent coughing and mild chest pain. The chest radiograph revealed a well delineated, solid lesion of the left upper lobe (Figure 5 and 6). For further characterization of this lesion, a contrast-enhanced CT examination was performed (Figure 7 and 8). Due to the location of the lesion and the characteristic fat attenuation, a diagnosis of subpleural lipoma was made. The radiologist, the general practitioner and the pulmonologist agreed that the mild chest pain and the intermittent coughing that our patient had reported before the examination were caused by recurrent attacks of bronchitis.

## Discussion

Before the implementation of computed tomography in diagnostic medical imaging, most subpleural lipomas were detected during autopsies or incidentally during thoracic surgery. Today, if a pathological pleural or pulmonal lesion is detected on a chest radiograph, CT is a very elegant method to quantify and characterize these lesions. In cases of subpleural lipoma, which shows typical fat attenuation values of approximately -50 to -100HU (Hounsfield units), the diagnosis of a fat-containing tumor of the pleura can be made. However, making the differentiation between well differentiated, malignant liposarcomas and benign lipomas may be challenging on CT images. The typical characteristics of a malignant tumor include invasive growth, inhomogeneous enhancement after intravenous contrast medium application, poor delineation of the lesion and the occurrence of metastases. In the case of lipomas and liposarcomas, even for experienced histopathologists it is a challenge to differentiate between these two tumor entities since the only key finding might be the mitosis rate of the tumor cells [2]. However, CT is able to depict potential invasion of adjacent structures such as the ribs or the soft tissue of the chest wall as well as potential enhancement after the application of contrast agent, although rib erosion has also been reported to occur with benign subpleural lipomas due to the local mass lesion [3]. Benign lipomas usually show no contrast enhancement. The locations of the lipomas presented in this case report are not typical for these tumors, which account for the most common soft tissue tumors in humans and can occur wherever adipose tissue is present; thus, if a solid, well delineated pleural lesion is depicted on a chest radiograph, the differential diagnosis of a subpleural lipoma has to be taken into consideration.

## Conclusions

CT should be considered the imaging method of choice to elucidate the location and extent of subpleural lipomas.

## Competing interests

The authors declare that they have no competing interests.

## Authors' contributions

CAK had the initial idea for this work and was responsible for writing the manuscript. PS handled the patient data acquisition and the literature research. TF contributed to the editing of the manuscript. OFD was responsible for creating the figure files. SL contributed to the editing and writing of the manuscript. All authors read and approved the final version of the manuscript.

## Consent

Written informed consent was obtained from both patients for publication of this case report and any accompanying images. A copy of the written consent is available for review by the Editor-in-Chief of this journal.
